# Functional characterization of *Candida auris* DHA1 transporters conferring flucytosine resistance

**DOI:** 10.3389/fmicb.2026.1806653

**Published:** 2026-05-08

**Authors:** Sarita Malik, Amandeep Saini, Rosy Khatoon, Atanu Banerjee

**Affiliations:** 1 Amity Institute of Biotechnology, Amity University Haryana, Gurugram, India; 2 Amity Institute of Integrative Sciences and Health, Amity University Haryana, Gurugram, India

**Keywords:** 5-fluorocytosine, antifungal resistance, *Candida auris*, DHA1 transporter, efflux pump

## Abstract

*Candida (Candidozyma) auris* has recently been designated a critical priority pathogen by the World Health Organization (WHO) in its Fungal Priority Pathogens List (FPPL). With resistance now widespread against frontline antifungals and limited availability of alternative therapeutics, combination regimens incorporating the nucleoside analog flucytosine (5-fluorocytosine; 5-FC) have gained renewed attention. While transporter-mediated resistance is well established for azole antifungals, knowledge on the molecular basis underlying reduced susceptibility to 5-FC in *C. auris* remains limited. Here, we aimed to functionally characterize two *C. auris* DHA1 transporters and to evaluate whether their inhibition may modulate 5-FC efficacy. Building on earlier reports showing elevated expression of two major facilitator superfamily (MFS) transporters belonging to the Drug/H^+^ antiporter-1 (DHA1) family upon 5-FC exposure, we now provide functional evidence supporting their contribution to 5-FC resistance. Heterologous expression of these transporters, B9J08_002663 (CauQdr2) and B9J08_004113 (CauMdr1.2) in a *Saccharomyces cerevisiae* system resulted in a marked increase in 5-FC resistance, with a > 2-fold shift in IC₅₀ values compared to the host strain. Moreover, analysis of ligand-bound structural models revealed a conserved interaction niche, consistent with structural and functional data from major facilitator superfamily (MFS) proteins, particularly DHA1 transporters. Furthermore, we also identified clorgyline as a selective inhibitor of CauMdr1.2, capable of modulating 5-FC susceptibility. Collectively, our findings uncover a DHA1 transporter-mediated mechanism of 5-FC resistance in *C. auris*, expanding the functional repertoire of such proteins and providing potential new targets for antifungal intervention. The findings further provide a rationale for designing 5-FC–based combination strategies incorporating transporter inhibitors to enhance drug efficacy and counteract efflux-mediated resistance.

## 1 Introduction

Fungal infections, despite imposing a significant global disease burden and contributing to considerable morbidity and mortality, remain underrecognized in public health discourse (Kriegl et al., 2025). In 2022, the World Health Organization (WHO) released its first global priority list of fungal pathogens and placed *Candida albicans*, *Candida auris*, *Aspergillus fumigatus*, and *Cryptococcus neoformans* in the critical priority group (WHO, 2022; Tan et al., 2025). *C. auris*, since its first identification in 2009, has been responsible for several healthcare-associated infections across the globe (Eix and Nett, 2025). Its ability to persist on skin and healthcare settings poses a significant challenge for health care providers (Lockhart, 2019; Mishra et al., 2023; Eix and Nett, 2025). *C. auris* is also characterized by a high degree of antifungal resistance, further worsening the situation (De Gaetano et al., 2024). Six distinct *C. auris* clades have been identified to date: Clade I originating from South Asia, Clade II from East Asia, Clade III from Africa, Clade IV from South America, Clade V from Iran and the recently described clade VI, which was detected in Singapore and shows no specific geographical association (Suphavilai et al., 2024). Among all the clades, I, III, and IV are majorly responsible for invasive infection outbreaks and often found to be multidrug-resistant (Rybak et al., 2022). Among the antifungal agents used to treat invasive *Candida* infections, echinocandins are the preferred choice as resistance toward azoles and amphotericin B is significantly high (Rybak et al., 2022; Kriegl et al., 2025). Epidemiological data suggests that around 90% of the clinical isolates are resistant to azoles, about ~50% show resistance to amphotericin B, and only around 5% exhibit resistance toward echinocandins (Lockhart et al., 2016; Rybak et al., 2022). Importantly, multidrug resistance is frequently associated with *C. auris* (~30%) and evidence of both echinocandin and pan-drug resistance has also been reported (O’Brien et al., 2020; Jacobs et al., 2022; Rybak et al., 2022; Lyman et al., 2025).

Combination therapy serves as an attractive therapeutic option to tackle antifungal resistant *C. auris* infections (O’Brien et al., 2020; Zhu et al., 2020). For instance, combination treatment with micafungin and amphotericin B has been shown exert strong antifungal activity against *C. auris* (Jaggavarapu et al., 2020). Interestingly, combinatorial regimens incorporating the nucleoside analog flucytosine (5-FC) also demonstrate strong promise (O’Brien et al., 2020; John et al., 2023).

**Figure 1:**
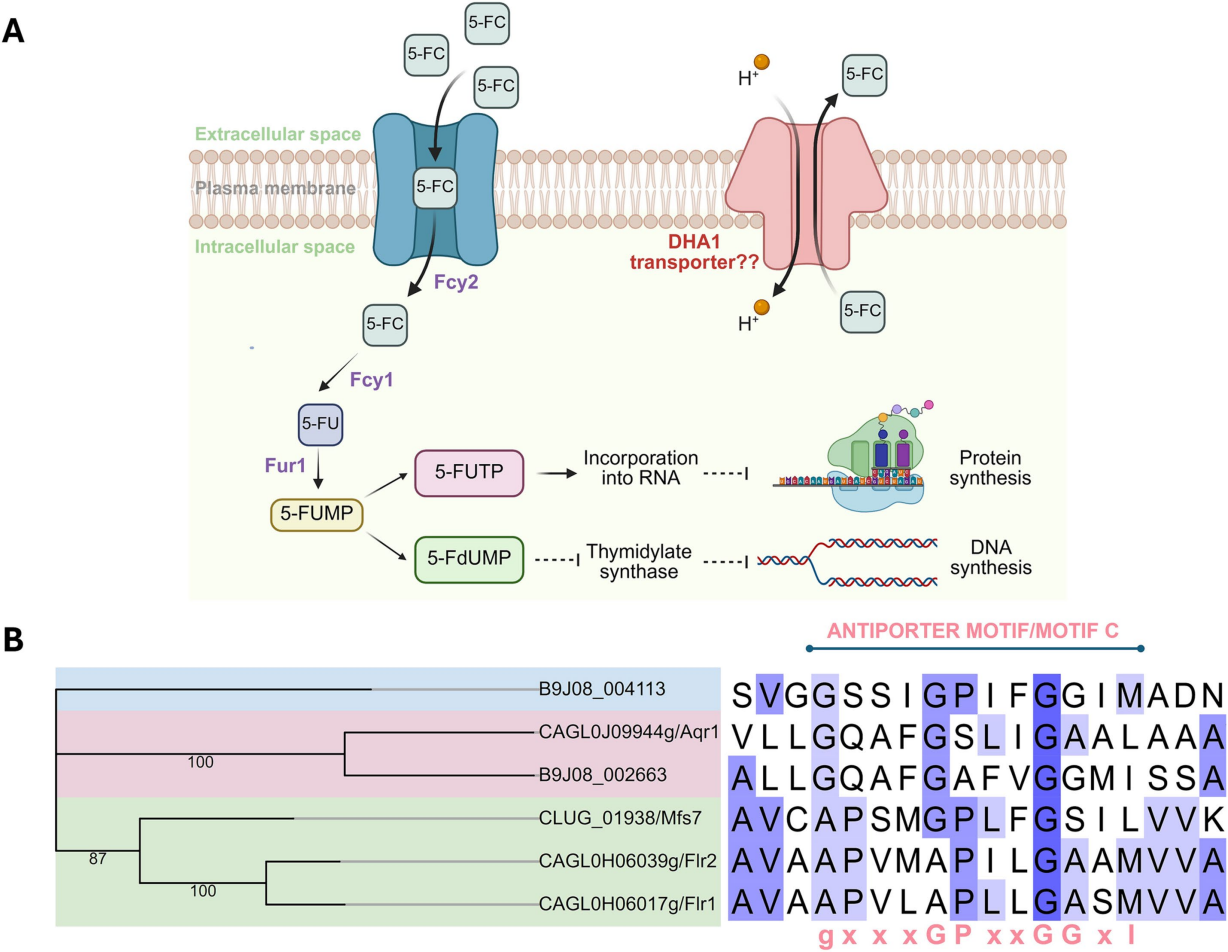
Pathway for flucytosine (5-FC) transport and metabolism, and phylogenetic analysis of transporters implicated in 5-FC resistance. **(A)** Schematic representation of 5-FC uptake, intracellular metabolism, and the proposed role of DHA1 transporters in 5-FC efflux. The putative DHA1 transporter is depicted as a proton-coupled antiporter, potentially mediating 5-FC efflux and thereby reducing intracellular drug accumulation. Created in BioRender (RRID:SCR_018361) Banerjee, A. (2026). Available online at: https://BioRender.com/1o7sbtl. **(B)** Phylogenetic relationship (left) of selected DHA1 family members implicated or proposed to be involved in 5-FC resistance, including B9J08_002663 (CauQdr2) and B9J08_004113 (CauMdr1.2), with bootstrap values indicated at major nodes. Sequence alignment (right) of the corresponding transmembrane regions highlights the conserved antiporter motif/motif C (GX₃GPXXGGXI), characteristic of DHA1 transporters. Residues are shaded in blue according to the degree of conservation.

5-FC functions as a prodrug and is imported by the cytosine permease, Fcy2 into the cell. Once inside, a fungal-specific cytosine deaminase encoded by *FCY1* converts 5FC into the active antimetabolite 5-fluorouracil (5-FU) (Delma et al., 2021; Phan-Canh et al., 2025). This is subsequently processed by the uracil phosphoribosyl transferase (UPRT) encoded by *FUR1*, generating 5-fluoro-uridylate (5-FUMP). 5-FUMP once phosphorylated into 5-UTP, can be incorporated into the RNA and hamper protein synthesis. 5-FUMP can also be reduced to 5-fluoro-2′-deoxyuridylate (5-FdUMP), which inhibits the enzyme thymidylate synthetase, and consequently DNA synthesis (Figure 1A). While 5-FC monotherapy is less effective due to rapid development of resistance (Ramos et al., 2025), it is employed as part of combination therapy in clinical settings for the management of *C. auris* infections (Gohel, 2022).

Molecular mechanism of resistance to 5-FC is well-understood in fungal pathogens and typically involves loss-of-function mutations along the *FCY2*–*FCY1*–*FUR1* axis (Delma et al., 2021). In *C. auris*, specific mutations in *FUR1* and *FCY2* have been identified to be associated with 5-FC resistance in clinical isolates (Rhodes et al., 2018; Jacobs et al., 2022). A recent study exploited the adaptive laboratory evolution (ALE) approach to understand 5-FC resistance *in vitro* (Phan-Canh et al., 2025). The authors identified and confirmed mutational inactivation of *FUR1* as a determinant of 5-FC resistance. Furthermore, the authors also detected a novel indel mutation in *FCY2* in one of the adapted isolates. Interestingly, the authors reported 5-FC tolerance in an adapter that does not involve the *FCY2*–*FCY1*–*FUR1* axis. Transcriptomic analysis of this adapter revealed upregulation of several membrane transporter encoding genes. Notably, two of the upregulated genes were putative Drug/H^+^ antiporter-1 (DHA1) pump-encoding genes, B9J08_002663 and B9J08_004113 (Phan-Canh et al., 2025). While B9J08_002663 is a homolog of *C. albicans QDR2*, the closest homolog of B9J08_004113 is *MDR1*. Although this *C. auris* gene is referred to as *MDR1.2* in the scientific literature (Shivarathri et al., 2022; Phan-Canh et al., 2025), our previous phylogenetic analysis of *C. auris* DHA1 transporters showed that this protein lies outside the Mdr1/Flr1 cluster and displays homology to the *S. cerevisiae* Yhk8 protein (Khatoon et al., 2022b). While clinical evidence of DHA1 transporter mediated 5-FC resistance is available only from *Candida* (*Clavispora*) *lusitaniae*, where deletion of the transporter gene *MFS7* in a drug resistant clinical isolate was shown to compromise 5-FC resistance (Asner et al., 2015; Kannan et al., 2019), several DHA1 pump encoding genes have been identified as contributors to 5-FC resistance in *Candida glabrata* (*Nakaseomyces glabratus*). Deletion of CAGL0J09944g/*AQR1*, CAGL0H06017g/*FLR1*, and CAGL0H06039g/*FLR2* resulted in increased intracellular accumulation of ^3^H–5-FC in *C. glabrata* (Costa et al., 2013a; Pais et al., 2016). Given the importance of efflux-mediated antifungal resistance in *Candida* species and the observed upregulation of DHA1 genes, B9J08_002663 and B9J08_004113 in *C. auris* upon exposure to 5-FC, we sought to investigate the function of these two transporter proteins in mediating 5-FC resistance through heterologous expression in a transporter deficient *S. cerevisiae* strain. In this study, we show that both transporter genes can confer 5-FC resistance, further supported by ligand-bound structural models identifying a 5-FC binding pocket enriched in residues equivalent to those essential for *Candida albicans* Mdr1 function. Furthermore, we demonstrate that B9J08_004113, referred to in the literature as *MDR1.2*, does not contribute to resistance to antifungals/xenobiotics in a manner comparable to *MDR1*, B9J08_003981 (Khatoon et al., 2022b; Toepfer et al., 2023b); however, it is responsible for the synergistic interaction between 5-FC and clorgyline.

## 2 Materials and methods

### 2.1 Multiple sequence alignment and phylogenetic analysis

DHA1 protein sequences from *C. auris* and *C. glabrata* were retrieved from the *Candida* genome database (CGD), while the *C. lusitaniae MFS7* (CLUG_01938) protein sequence was retrieved from NCBI using the Genbank assembly GCA_009498055.1. Multiple sequence alignment (MSA) was performed using MAFFT (RRID:SCR_011811) (Katoh and Standley, 2013) and the alignment was inspected and visualized using Jalview (RRID:SCR_006459) (Waterhouse et al., 2009). The MSA was subsequently used to construct a phylogenetic tree using the IQ-TREE (RRID:SCR_017254) webserver which employs a maximum likelihood approach for tree inference (Trifinopoulos et al., 2016). The best-fit model of amino acid substitution, as determined by Bayesian Information Criterion (BIC) scores, was LG + G4, and branch support was assessed using 1,000 bootstrap replicates. The resulting consensus tree was exported in Newick format and visualized using iTOL (RRID:SCR_018174) (Letunic and Bork, 2021).

### 2.2 Structural analysis

Structural models of 5-FC bound transporter proteins were built using the three major open-source reproduction of AlphaFold3 (AF3), Boltz-2 (Passaro et al., 2025), Chai-1 (Discovery et al., 2024), and Proteinix (ByteDance AML AI4Science Team et al., 2025), which show good performance in mapping protein-ligand interactions (Xu et al., 2025). In these workflows, 5-FC was co-modeled during structure prediction, rather than introduced through independent docking. No explicit or implicit membrane restraints were applied during model generation. The major parameters used for comparisons included: predicted local distance difference test (pLDDT), predicted TM-score (pTM), interface pTM (ipTM). For B9J08_002663 (CauQdr2), the top-ranked models from Proteinix and Boltz-2 were used for analyses. However, for B9J08_004113 (CauMdr1.2), while the top model of Proteinix was retained, the 5th-ranked Boltz-2 model was selected, because it displayed an improved (0.91 vs. 0.88) ipTM score, providing greater confidence in the predicted interface between the transporter and 5-FC. The selected structure models were analyzed and visualized using Chimera-X (RRID:SCR_004097) (Pettersen et al., 2021).

### 2.3 Materials

All routine chemicals used were purchased from Sigma-Aldrich Co. (St. Louis, MO) and SRL Pvt. Ltd., Mumbai, India. Fluconazole (FLC), clotrimazole (CTR), voriconazole (VRC), amphotericin B (AMB), Clorgyline (CLO), anidulafungin (AFG), benomyl (BEN), cycloheximide (CHX), anisomycin (ANI), 4-nitroquinoline1-oxide (4-NQO), Quinidine sulfate and dimethyl sulfoxide (DMSO) were procured from Sigma-Aldrich Co. (St. Louis, MO). Flucytosine (5-FC) and posaconazole (POS) were purchased from Tokyo Chemical Industry Co., Ltd. (TCI), Tokyo, Japan. Oligonucleotides were purchased from Barcode Biosciences, Bengaluru, India and are listed in the Supplementary Table S1. All materials used for sodium dodecyl sulfate-polyacrylamide gel electrophoresis (SDS-PAGE) and immunoblotting, except for protein molecular weight marker and antibodies, were purchased from Bio-Rad Laboratories, Inc. (CA, United States). The protein molecular weight marker was purchased from NextGen Life Sciences (Delhi, India) and the anti-GFP monoclonal antibody was purchased from Santa Cruz Biotechnology Inc. (Texas, United States). The fungal protease inhibitor ProteaseArrest™ and Bicinchoninic Acid (BCA) protein estimation kit were purchased from G-Biosciences, MO, United States.

### 2.4 Strains and culture conditions

The *C. auris* CBS10913T strain was obtained from the CBSKNAW fungal culture collection and reported in our previous study (Khatoon et al., 2022b). Routine culturing and maintenance of the yeast strains was done in the YEPD medium (containing yeast extract, peptone, and dextrose) from HiMedia Laboratories, Mumbai, India. The *S. cerevisiae* transformants were selected on growth medium lacking uracil. The medium consisted of 0.67% yeast nitrogen base (YNB) without amino acids (Difco, Becton, Dickinson and Company, Sparks, MD, United States), 0.2% dropout mix lacking uracil (Sigma-Aldrich Co.), and 2% glucose (HiMedia). Plasmids were maintained in *Escherichia coli* DH5α (Thermo Fisher Scientific, Waltham, MA, United States) and cultured in Luria–Bertani medium (HiMedia) supplemented with 100 μg/mL ampicillin (Amresco, Solon, United States). All the yeast strains used or constructed in this study are listed in the Supplementary Table S2.

### 2.5 Molecular cloning of *Candida auris* genes B9J08_002663 and B9J08_004113 in pABC3-*GFP* and overexpression in AD1-8u^−^

Genes with IDs B9J08_002663 and B9J08_004113 were amplified from genomic DNA of the CBS10913T strain with forward and reverse primers harboring *Pac*I and *Not*I restriction sites, respectively, and cloned in the pABC3-GFP vector (Lamping et al., 2007) as described previously (Khatoon et al., 2022b). For expression in *S. cerevisiae*, the plasmids were digested with *Asc*I to release the transformation cassette and introduced into AD1-8u^−^ cells using the lithium acetate–based chemical transformation method. Transformants were selected on SD-Ura^−^ medium and positive colonies were identified using gene-specific PCR, fluorescence microscopy, and DNA sequencing.

### 2.6 Confocal microscopy

For microscopy, exponential phase cells of constructed overexpression strains were washed with 1X PBS buffer before being examined under the confocal microscope (Model:- A1R HD 25; Nikon Instrument Inc., Melville, NY, United States) with a 60X oil immersion objective lens.

### 2.7 Small-scale plasma membrane preparations and immunodetection of GFP-tagged proteins

Yeast plasma membrane (PM) fractions were isolated using a small-scale protocol adapted from (Madani et al., 2021). Briefly, a single colony was pre-cultured in 10 mL YEPD medium at 30 °C with shaking for 7–8 h. This 10 mL pre-culture was next added to 40 mL of fresh YEPD medium followed by overnight growth of approximately 16 h with OD_595_ approximately reaching 3–4. Cells corresponding to ~40 OD units were harvested, washed with ice-cold sterile water, and resuspended in 0.5 mL homogenization buffer (50 mM Tris–HCl pH 7.5, 0.5 mM EDTA, 20% glycerol) supplemented with a fungal protease inhibitor cocktail (ProteaseArrest™; G-Biosciences). Next, cells were disrupted by vortexing with 0.5 mm silica beads (Sigma-Aldrich Co.) in 6 cycles with intermittently cooling on ice. The beads were settled by low-speed centrifugation, followed by carefully collecting the cell homogenate using pipette. Next, the cell homogenate was centrifuged at 5,156 × g for 5 min at 4 °C to remove cell debris, unbroken cells, and nuclei. The clarified homogenate (~300–400 μL) was diluted with ice-cold homogenization buffer containing protease inhibitor to make a total volume of 1.5 mL. Following this, the PMs were pelleted by high-speed centrifugation at 17,968 × g for 1 h at 4 °C. The PM pellets were finally resuspended in 100 μL homogenization buffer, and protein concentration was determined using the BCA assay kit (G-Biosciences). The membrane preparations were either used immediately or stored at −80 °C.

PM preparations (5 μg protein) were resolved on 10% SDS–PAGE stain-free gels prepared using Bio-Rad TGX Stain-Free™ FastCast™ acrylamide solutions. Following electrophoresis, the gel was vertically bisected; one half was used for immunoblotting, while the other half was imaged to visualize total protein using a Bio-Rad ChemiDoc XRS + system. Proteins were transferred onto polyvinylidene fluoride (PVDF) membranes using the Trans-Blot Turbo system (Bio-Rad). Membranes were blocked with blotting-grade blocker (Bio-Rad) and probed with HRP-conjugated anti-GFP monoclonal antibody (Santa Cruz Biotechnology; Cat# sc-9996 HRP, RRID:AB_3713277) at a 1:5000 dilution to detect C-terminal GFP-tagged transporter proteins. Signals were developed using Clarity Western ECL substrate (Bio-Rad) and imaged on an Amersham Imager 680 system.

### 2.8 Drug sensitivity assessments

All screening assays were performed using a synthetic defined (SD) medium. This medium comprised 0.67% YNB without amino acids, 0.2% yeast synthetic dropout supplement without uracil, 0.002% uracil, and 2% glucose. To prevent masking 5-FC toxicity in the ura^−^ background, all assays were performed under uracil-limiting conditions (0.002%), which permitted basal growth while maintaining 5-FC sensitivity in the parental strain.

#### 2.8.1 Broth microdilution assay

The strains were tested for susceptibility to different antifungal drugs using a modification of the CLSI M27-A3 broth microdilution reference method, as the AD1-8u^−^ strain does not grow in the RPMI medium used in the CLSI method (Holmes et al., 2012). For the broth microdilution (BMD) assay, the cells were diluted in 0.9% saline solution from freshly patched plates and OD_595_ was set to 0.1. Next, serial two-fold dilutions for each compound were prepared in SD media and incubated with log-phase cells (~10^4^ cells/ml) at 30 °C for 48 h. After 48 h, growth was quantified by measuring the optical density was recorded at 595 nm using a microplate reader (iMark™ Microplate Absorbance Reader-BioRad). The 5-FC dose–response curves were generated by plotting percent inhibition against the logarithm of inhibitor concentration and analyzed by non-linear regression using a four-parameter logistic (4PL) model with a variable Hill slope in GraphPad Prism v8.4.3 (RRID:SCR_002798). The model was fit without constraints to estimate the minimum response (bottom), maximum response (top), log (half-maximal inhibitory concentration; IC₅₀), and Hill slope. Confidence intervals (95%) for fitted parameters were determined using profile likelihood method. The statistical differences between the IC₅₀ values of host and overexpression strains were evaluated using an extra sum-of-squares F-test by comparing non-linear regression models with shared versus independent LogIC₅₀ parameters.

#### 2.8.2 Serial dilution spot assay

For serial dilution spot assay, yeast suspensions from freshly patched YEPD plates were prepared in 0.9% NaCl solution and serially diluted five-fold. A 3 μL aliquot from each dilution was spotted onto SD agar plates with or without 5-FC (4 μg/mL) and incubated for 48 h at 30 °C. After 48 h, images of the plates were acquired using Bio-Rad gel documentation system.

#### 2.8.3 Growth kinetics

The growth kinetics assay was performed by a microcultivation method in a 96-well plate using liquid handling system (Tecan, Austria) in SD medium at 30 °C. Briefly, overnight cultures were diluted to an initial OD_595_ of 0.1, and 200 μL of each culture, in the presence or absence of 4 μg/mL 5-FC, was dispensed into the wells of a 96-well plate. Optical density was recorded at 595 nm at 30-min intervals over a period of 48 h.

#### 2.8.4 Checkerboard synergy assay

The synergy between 5-FC and CLO was evaluated using a checkerboard microdilution assay followed by Bliss independence analysis. Herein, two-fold serial dilutions of 5-FC and CLO were prepared in SD medium and dispensed into 96-well microtiter plates such that concentration gradients of 5-FC and CLO were arranged along the abscissa and ordinate, respectively. Yeast cultures from freshly patched plates were diluted in fresh medium to a final inoculum of approximately (~10^4^ cells/ml), and 100 μL of this cell suspension was added to each well. Wells without drug served as growth controls. Plates were incubated at 30 °C for 48 h. Fungal growth was quantified by measuring optical density at 595 nm using a microplate reader. Percent inhibition for each condition was calculated relative to the untreated control. Synergy scores were calculated using the Bliss independence model available in SynergyFinder 3.0 (RRID:SCR_026127) (Ianevski et al., 2022). The base-line correction was applied to minimize the impact of problematic data points in deriving synergy scores. The assays for both overexpression strains were performed in three biological replicates, and inhibition data from all three measurements were used to calculate Bliss synergy scores and 95% confidence intervals (95% CI). Bliss synergy scores were interpreted using the default SynergyFinder thresholds, where scores < −10 indicate antagonistic interactions, scores between −10 and 10 indicate additive effects, and scores > 10 indicate synergistic interactions. The same interpretation was also applied to the most synergistic area (MSA) score, which represents the most synergistic 3-by-3 dose-window in a dose–response matrix.

## 3 Results

### 3.1 Comparative sequence analysis of B9J08_002663 and B9J08_004113 with 5-FC transporter proteins of *Candida*

In view of the observed upregulation of the two DHA1 genes, B9J08_002663 and B9J08_004113, in 5-FC tolerant strains (Phan-Canh et al., 2025), at first, we investigated their sequence-level relatedness to known 5-FC DHA1 transporter proteins from other *Candida* species (Costa et al., 2013a; Asner et al., 2015; Pais et al., 2016). We performed a multiple sequence alignment (MSA) of the corresponding protein sequences to confirm their status as DHA1 pumps based on the presence of the conserved antiporter motif: GxxxGPxxGGxI (Sharma et al., 2022). It was interesting to note that while this signature sequence was preserved across all analyzed sequences, several notable atypical substitutions were detected at key positions in many sequences, including those in the proteins encoded by B9J08_002663 and B9J08_004113. For instance, the conserved proline is replaced by alanine in B9J08_002663 and the isoleucine is replaced by methionine in B9J08_004113 (Figure 1B).

To understand the evolutionary relationships among these transporter proteins, we also carried out a phylogenetic analysis. The protein encoded by B9J08_002663 clustered closely with *C. glabrata* Aqr1 (Figure 1B). Notably, besides being a homolog of *C. albicans* Qdr2, the B9J08_002663-encoded protein also shares close homology with Aqr1 from *S. cerevisiae* and *C. glabrata* (Tenreiro et al., 2002; Vargas et al., 2004; Costa et al., 2013a; Khatoon et al., 2022b). Based on these relationships, we refer to the B9J08_002663 protein as CauQdr2 throughout this study. The *C. glabrata* transporters Flr1 and Flr2 also clustered together and formed part of a larger clade that included *C. lusitaniae* Mfs7 (Figure 1B). Since all three proteins are closely related to Mdr1, their clustering was expected. Interestingly, the B9J08_004113 protein appeared as an outgroup (Figure 1B). Despite sharing homology with Mdr1, we have previously shown that it is not part of the same cluster as Mdr1/Flr1 group of *C. albicans*, *C. glabrata*, and *S. cerevisiae* DHA1 pumps (Khatoon et al., 2022b). However, since the B9J08_004113 gene is referred to as *MDR1.2* in literature, we designate the corresponding protein as CauMdr1.2.

### 3.2 Subcellular localization studies of CauQdr2 and CauMdr1.2

**Figure 2:**
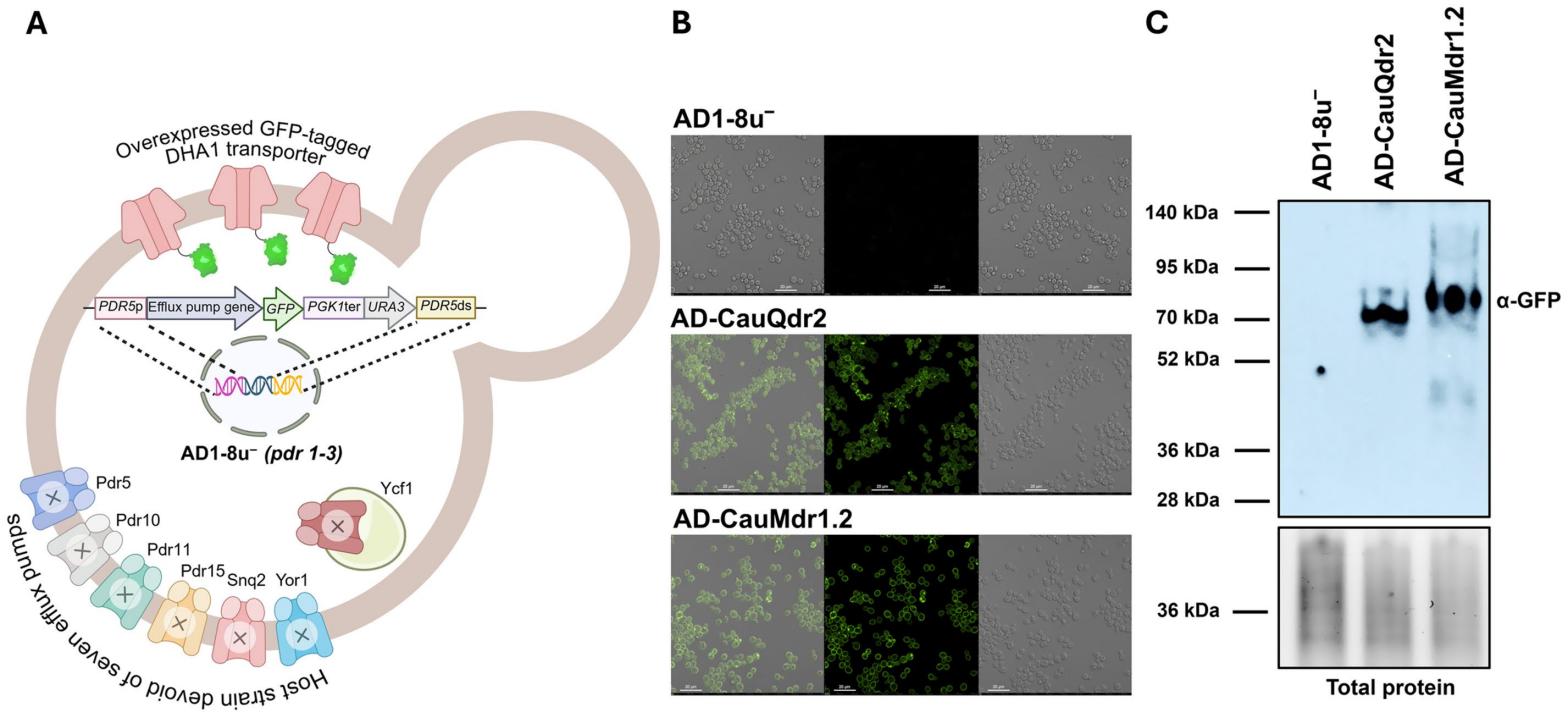
Heterologous expression and membrane localization of GFP-tagged DHA1 transporters in the AD1-8u^−^ strain. **(A)** Schematic representation of the heterologous overexpression system used for DHA1 transporters in the *Saccharomyces cerevisiae* AD1-8u^−^ strain, which lacks major endogenous efflux pumps. GFP-tagged CauQdr2 and CauMdr1.2 were expressed under the control of the *PDR5* promoter, enabling visualization and functional analysis of transporter localization and activity. Created in BioRender (RRID:SCR_018361) Banerjee, A. (2026). Available online at: https://BioRender.com/1o7sbtl. **(B)** Confocal fluorescence microscopy images showing cellular localization of GFP-tagged transporters. Bright-field, GFP fluorescence, and merged images (right to left) are shown. **(C)** Western blot analysis of plasma membrane (PM) preparations confirming expression of GFP-tagged CauQdr2 and CauMdr1.2 in the respective overexpression strains. PM protein (5 μg per lane) was resolved by SDS-PAGE and probed with an anti-GFP monoclonal antibody. For loading control, the gel was vertically bisected post-electrophoresis; the bottom panel displays a representative portion of the corresponding stain-free gel to visualize the total protein profile.

To study the functional attributes of the two proteins, we expressed them as C-terminal GFP-tagged proteins in the AD1-8u^−^ strain. This host strain lacks seven major antifungal drug transporters and carries the *pdr1-3* gain of function mutation which drives constitutive hyperactivation of the *PDR5* promoter, under whose control we place our genes of interest (Lamping et al., 2007) (Figure 2A). This overexpression system and its derivatives have been extensively utilized to map the substrates of a large spectrum of fungal efflux pumps, including CauMdr1 (Khatoon et al., 2022b; Toepfer et al., 2025; Zhao et al., 2025). The resulting overexpression strains were designated AD-CauQdr2 and AD-CauMdr1.2. We first employed confocal microscopy to examine the subcellular localization of the expressed proteins. As shown in the confocal microscopy images in Figure 2B, both CauQdr2 and CauMdr1.2 localized predominantly to the plasma membrane (PM). Notably, in AD-CauMdr1.2, majority of the fluorescence signals were restricted to the cell surface. However, in the AD-CauQdr2 strain, additional intracellular signals were detected, consistent with proteins being trapped within the intracellular structures (Figure 2B).

To further validate the PM localization, we isolated plasma membrane fractions from the AD1-8u^−^, AD-CauQdr2 and AD-CauMdr1.2 strains and probed them with anti-GFP antibody. Consistent with the observations made using microscopy, immunoblot analysis confirmed the presence of both proteins in the PM (Figure 2C). However, the proteins were found to migrate at lower molecular weights than expected. Based on the primary sequences, the expected size for CauQdr2-GFP and CauMdr1.2-GFP are ~87 kDa and ~91 kDa, respectively. However, both proteins exhibited an apparent reduction of ~10–15 kDa on SDS-PAGE. Importantly, no additional lower-molecular-weight bands were observed, suggesting that the shift is unlikely to result from proteolytic degradation. A similar difference in the migration pattern on SDS-PAGE was also seen in the case of CauMdr1 by Toepfer and colleagues (Toepfer et al., 2023b). While such anomalous mobility of many other PM proteins is well-documented (Rath and Deber, 2013), additional analyses including size-exclusion chromatography of the purified proteins are warranted to confirm their respective molecular weights.

### 3.3 Drug-susceptibility profiling of CauQdr2 and CauMdr1.2 overexpression strains

To assess the ability of CauQdr2 and CauMdr1.2 to confer resistance to antifungal drugs, we performed broth microdilution assay for the host AD1-8u^−^ strain, and AD-CauQdr2 and AD-CauMdr1.2 strains against 5-FC and other antifungal compounds including azoles, an echinocandin and a polyene. The selected panel of antifungal drugs included: 5-FC, fluconazole (FLC), clotrimazole (CTR), voriconazole (VRC), posaconazole (POS), anidulafungin (AFG) and amphotericin-B (AMB). As shown in Table 1, we could detect 4-fold change in MIC values of 5-FC against both AD-CauQdr2 and AD-CauMdr1.2 strains compared to the host strain. For other antifungal drugs, we did not notice any significant change in the MIC values (Table 1).

**Table 1:** Minimum inhibitory concentration (MIC) values of antifungal agents determined by broth microdilution assays in SD medium.

Strains	MIC [μg/ml]						
	5-FC	FLC	VRC	CTR	POS	AFG	AMB
AD1-8u^−^	8	0.5–1	0.004–0.008	0.015–0.031	<0.125	>2	0.062
AD-CauQdr2	32	0.5	0.004–0.008	0.015–0.031	<0.125	>2	0.062
AD-CauMdr1.2	32	1	0.004–0.008	0.015	<0.125	>2	0.062

The MIC was defined as the lowest drug concentration at which no visible growth was observed. Values represent the range of MICs observed across three independent experiments. When the MIC varied across the three experiments, the full range is reported, when all experiments yielded the same result, a single value is presented.

**Figure 3:**
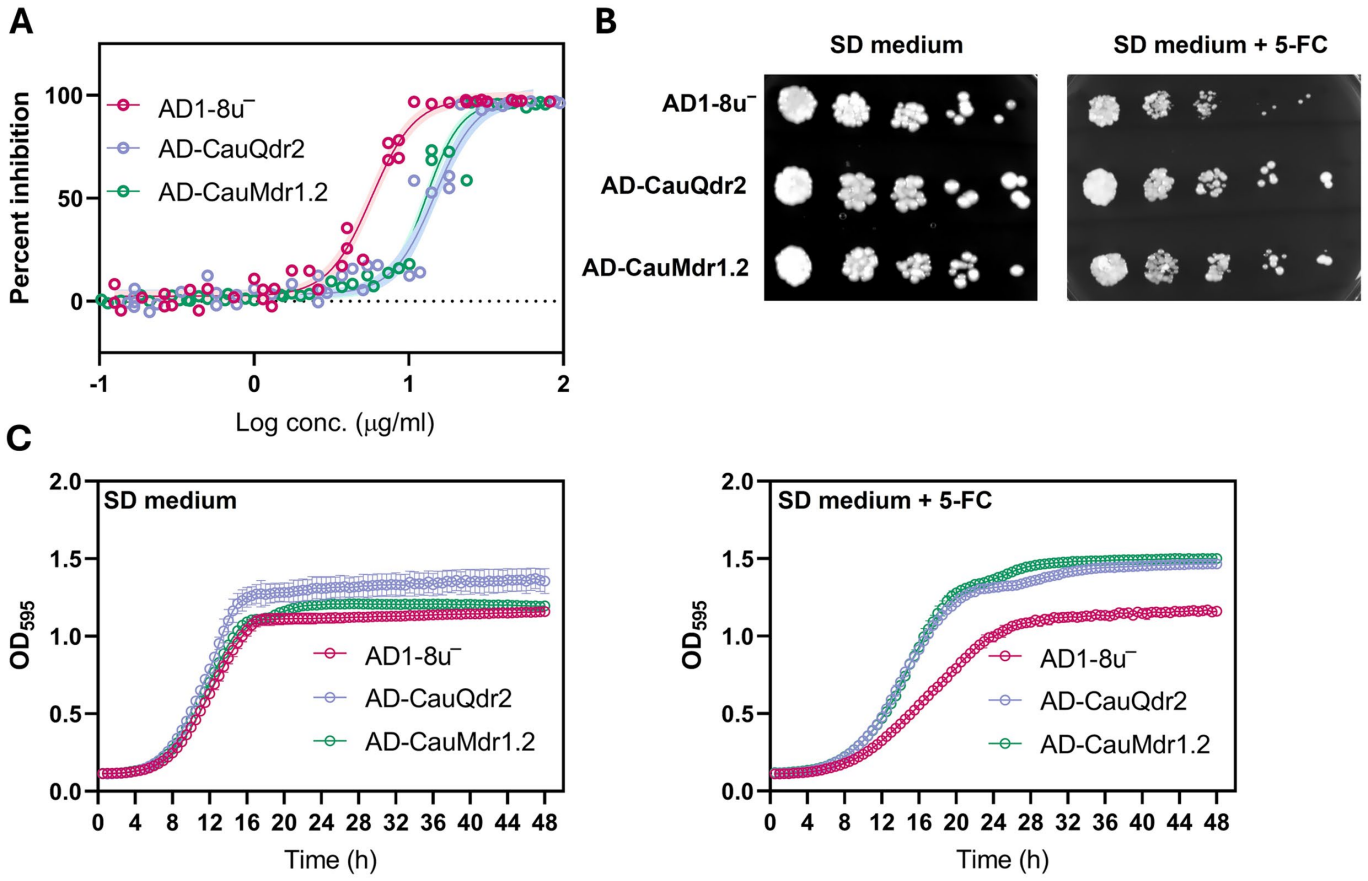
Flucytosine (5-FC) susceptibility profiling of DHA1 transporter overexpression strains. **(A)** Dose–response curves showing percent growth inhibition of the parental AD1-8u^−^ strain and strains overexpressing CauQdr2 (AD-CauQdr2) or CauMdr1.2 (AD-CauMdr1.2) across increasing concentrations of 5-FC. Experiments were performed using four independent biological replicates. Data are plotted as percent inhibition relative to untreated controls; shaded regions represent the 95% confidence intervals (CI). **(B)** Serial dilution spot assays comparing growth of AD1-8u^−^, AD-CauQdr2, and AD-CauMdr1.2 strains on SD medium in the absence or presence of 4 μg/mL 5-FC. **(C)** Growth kinetics of the host and overexpression strains monitored as OD_595_ in SD medium alone (left) or SD medium supplemented with 4 μg/mL 5-FC (right). Data represent mean ± standard deviation from three independent experiments.

To quantitatively assess the magnitude of 5-FC resistance conferred by CauQdr2 and CauMdr1.2, we generated dose response curves for all three strains and determined their IC_50_ values (Figure 3A). The results revealed marked differences (2.2–2.5-fold) in 5-FC susceptibility between the host strain and the two strains overexpressing the DHA1 pumps. While the AD1-8u^−^ strain exhibited an IC_50_ value of 5.74 μg/mL (95% CI: 5.36–6.15), overexpression of transporter CauQdr2 resulted in a significantly higher IC_50_ of 14.73 μg/mL (95% CI: 13.75–15.78), indicative of decreased drug susceptibility. A similar observation was also made for CauMdr1.2, which showed an IC_50_ of 12.88 μg/mL (95% CI: 12.35–13.41). The extra sum-of-squares F-test confirmed that the Log IC_50_ values of both overexpression strains differed significantly from the host strain (*p* < 0.0001). Overall, the results confirmed transporter-dependent increase in resistance toward 5-FC.

We further evaluated the effect of 5-FC on the three strains using solid agar spot assay and growth kinetics profiling at sub-IC_50_ concentration of 5-FC for AD1-8u^−^. As shown in Figure 3B, inhibitory effect of 5-FC was evident in the case of the host strain, while both overexpression strains did not show any noticeable reduction in growth under the same condition. Consistent with these observations, growth kinetics measurements for 48 h revealed that AD-CauQdr2 and AD-CauMdr1.2 strains exhibited increased resistance toward 5-FC compared to the host strain (Figure 3C), reiterating that overexpression of the two transporters confers 5-FC resistance.

**Figure 4:**
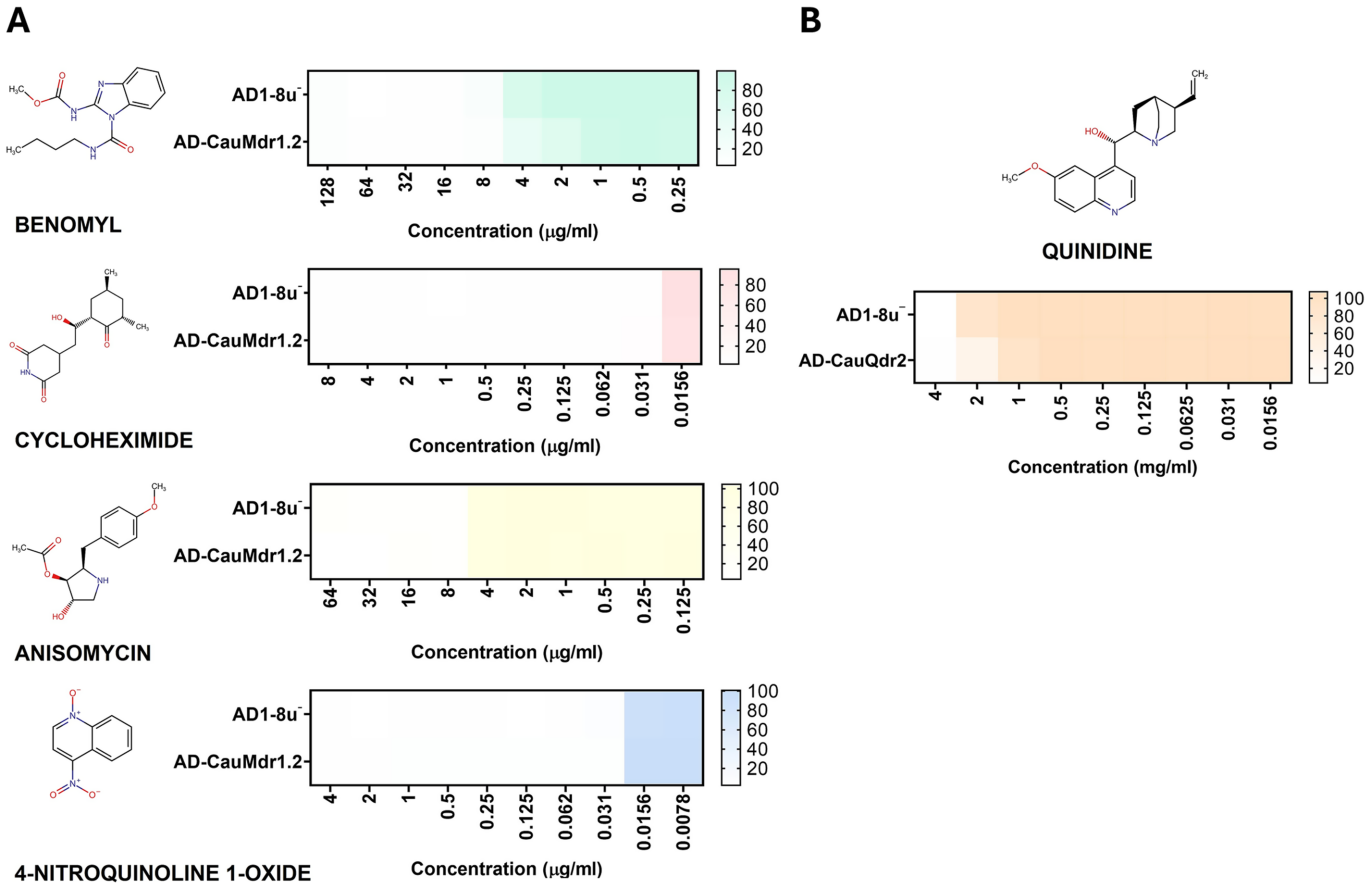
Substrate specificity profiling of CauMdr1.2 and CauQdr2 using broth microdilution assays. **(A)** Drug susceptibility profiles of the parental AD1-8u^−^ strain and the CauMdr1.2 overexpression strain (AD-CauMdr1.2) against established Mdr1 substrates. **(B)** Drug susceptibility profile of the parental AD1-8u^−^ strain and the CauQdr2 overexpression strain (AD-CauQdr2) against quinidine, a characteristic Qdr family substrate. Heat maps represent relative growth inhibition across increasing drug concentrations, with growth of untreated samples set to 100%. Structures were drawn using Chemspace.

While we observed that CauMdr1.2 and CauQdr2 did not confer any resistance to antifungal compounds except 5-FC, we further evaluated the growth patterns of the corresponding overexpression strains against xenobiotic substrates characteristic of each transporter class. For CauMdr1.2, we tested a panel of substrates known to be effluxed by Mdr1 of *C. albicans* and *C. auris*, including benomyl, cycloheximide, anisomycin, and 4-nitroquinoline 1-oxide (Redhu et al., 2016b; Khatoon et al., 2022b). However, no significant difference in growth was observed between the host strain and the AD-CauMdr1.2 strain across the range of concentrations tested (Figure 4A). For CauQdr2, we examined growth in the presence of quinidine, a hallmark substrate of the Qdr transporters (Vargas et al., 2004; Costa et al., 2013b) for evaluating the growth patterns. Contrary to the expected increase in resistance of the overexpression strain, the AD-CauQdr2 strain instead exhibited marginally increased sensitivity to quinidine compared with the host strain (Figure 4B).

### 3.4 Synergy analysis of flucytosine and clorgyline in transporter-overexpressing strains

**Figure 5:**
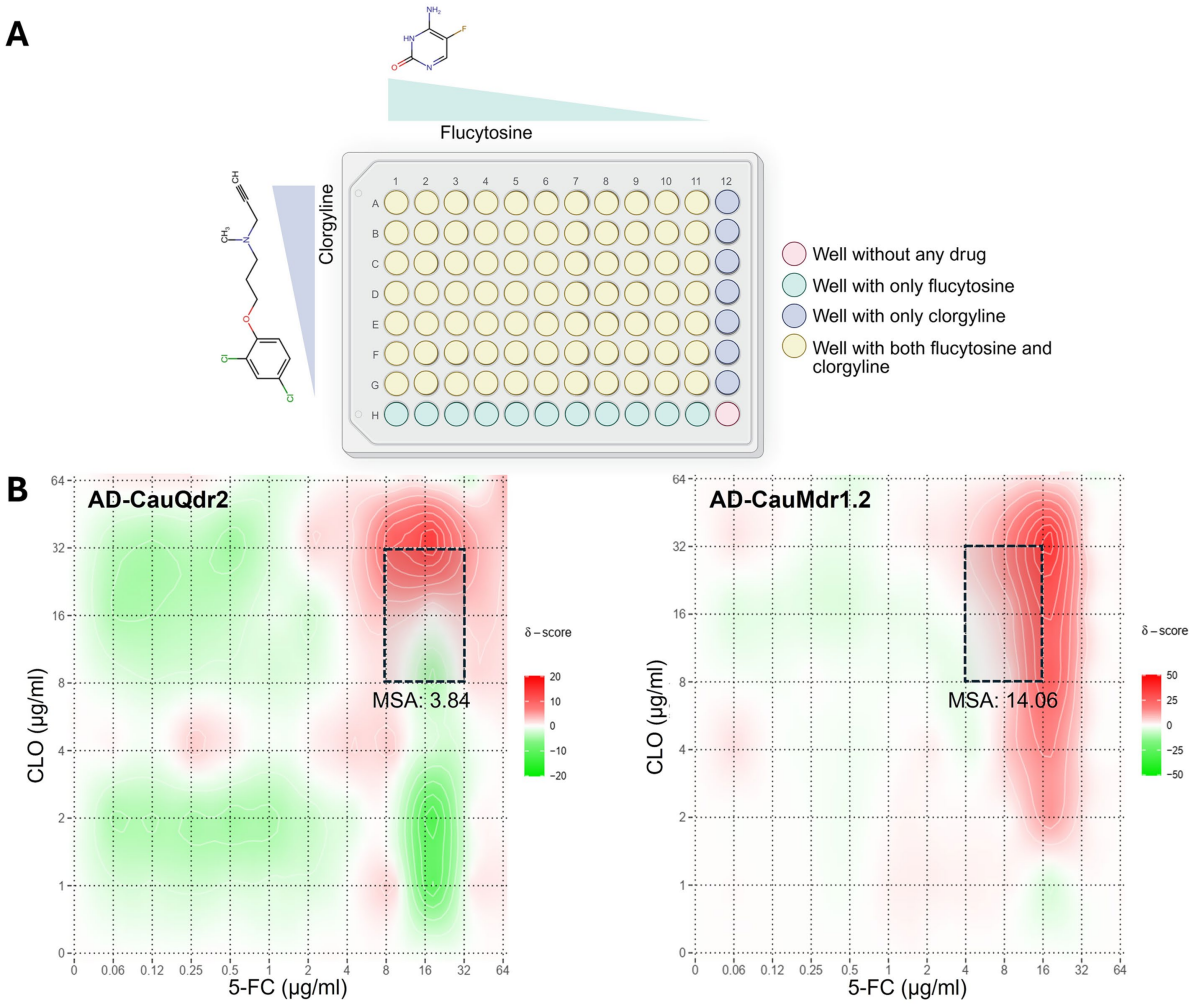
Synergistic interaction between flucytosine (5-FC) and clorgyline (CLO) in DHA1 transporter overexpression strains. **(A)** Schematic representation of the checkerboard assay used to evaluate drug–drug interactions between 5-FC and CLO. Serial dilutions of 5-FC and CLO were combined across a concentration matrix, and growth inhibition was quantified. Wells containing individual drugs or their combinations are indicated. Created in BioRender (RRID:SCR_018361) Banerjee, A. (2026). Available online at: https://BioRender.com/1o7sbtl. **(B)** Bliss independence synergy heat maps illustrating the interaction between 5-FC and CLO in AD-CauQdr2 and AD-CauMdr1.2 strains. Checkerboard assays were performed in three independent biological experiments. Synergy scores were calculated using SynergyFinder based on data pooled from all the experiments. The most synergistic area (MSA) is highlighted by dashed boxes, with the corresponding MSA score indicated.

Given that inhibition of efflux pumps is a prominent strategy to circumvent efflux-mediated antifungal resistance (Holmes et al., 2016), we next examined whether the broad spectrum efflux pump inhibitor CLO could sensitize the overexpression strains to 5-FC. Notably, CLO and its structural analogs have been earlier shown to inhibit Mdr1 and other major efflux pumps belonging to the ATP-binding cassette (ABC) superfamily in fungi, including *C. auris* (Holmes et al., 2012; Sastré-Velásquez et al., 2022; Toepfer et al., 2023a). We utilized the conventional checkerboard-based assay (Figure 5A) and quantified synergy between 5-FC and CLO using the Bliss independence model in SynergyFinder (Ianevski et al., 2022). Given that 5-FC and CLO act through independent targets and pathways, and their mechanisms of growth inhibition are pharmacologically distinct, the assumption of independent drug action underlying the Bliss model is biologically appropriate and more suitable than the Loewe additivity model. Synergy scores were interpreted using SynergyFinder default thresholds (>10 synergistic; −10 to +10 additive; <−10 antagonistic).

The AD-CauQdr2 strain displayed predominantly additive interactions across the concentration matrix, and the most synergistic area (MSA) score was 3.84. Of note, most of the synergy scores fell within the −10 to +10 window, indicating an overall additive effect (Figure 5B; left panel). In contrast, the AD-CauMdr1.2 overexpression strain exhibited a distinct and robust synergy hotspot at higher concentrations of CLO combined with mid-range doses of 5-FC (Figure 5B; right panel). This region, with an MSA of 14.06, exceeded the synergy threshold of +10. The magnitude of the synergy scores in this area indicated a transporter-dependent enhancement of 5-FC activity in the presence of CLO. Consistent with this observation, an approximately 1.5-fold reduction in the 5-FC IC₅₀ was observed at 16–32 μg/mL CLO. Altogether, these results suggest that CLO has a plausible inhibitory effect on CauMdr1.2-associated resistance to 5-FC, thereby sensitizing the AD-CauMdr1.2 strain to lower concentrations of 5-FC.

### 3.5 Structural modeling of 5-FC interactions with DHA1 transporters

**Figure 6:**
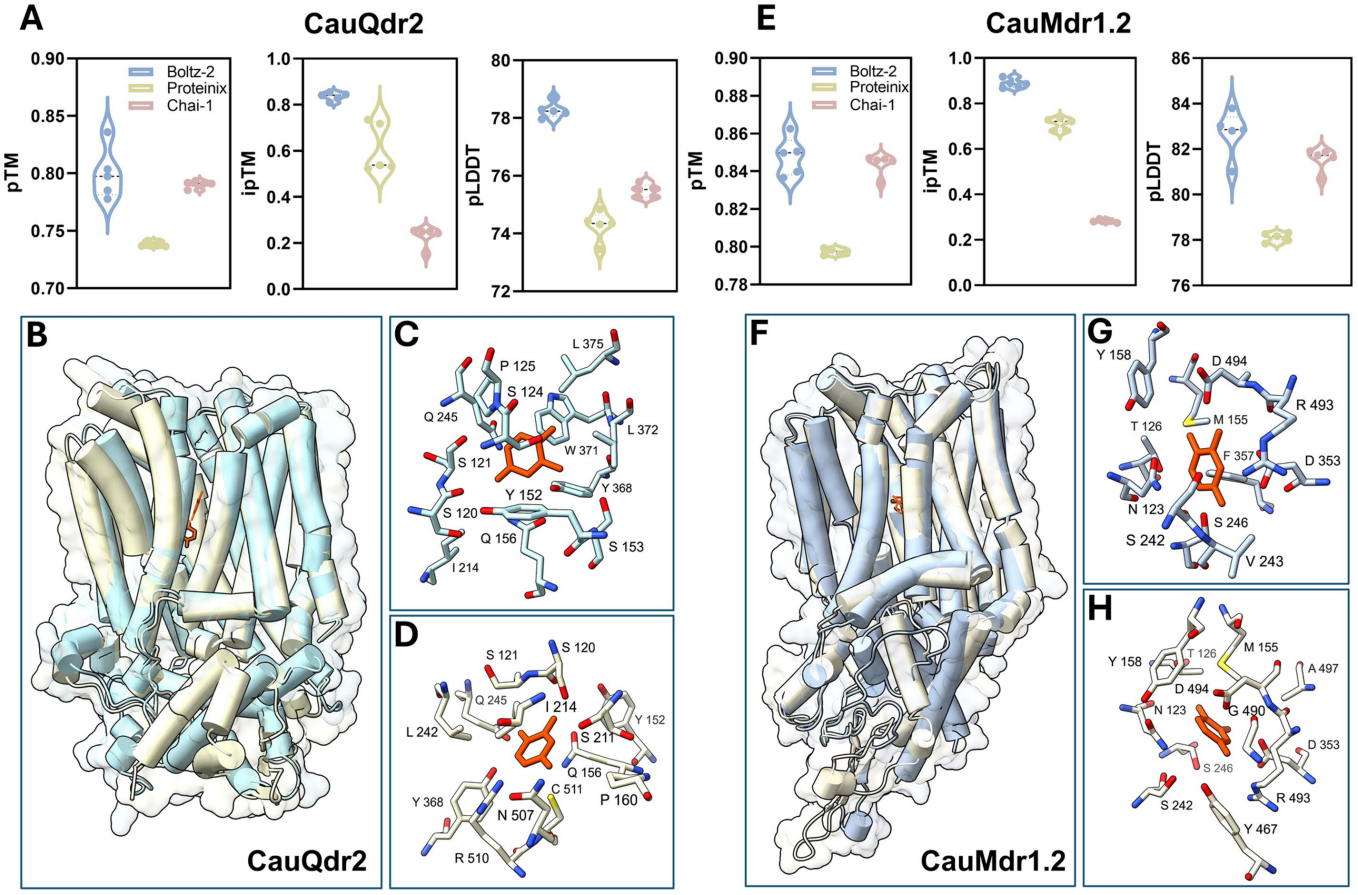
Structural modeling and analysis of flucytosine (5-FC) interaction sites in CauQdr2 and CauMdr1.2. **(A,E)** Violin plots showing model quality metrics (pTM, ipTM, and pLDDT) for CauQdr2 **(A)** and CauMdr1.2 **(E)** generated using Boltz-2, Proteinix, and Chai-1. **(B,F)** Superimposed structural models of CauQdr2 **(B)** and CauMdr1.2 **(F)** displaying the characteristic 12-transmembrane helix architecture of DHA1 transporters. For CauQdr2, Boltz-2 and Proteinix models are shown in powder blue and light goldenrod yellow colors, respectively. For CauMdr1.2, Boltz-2 and Proteinix models are shown in light steel blue and cornsilk, respectively. **(C,D)** Predicted 5-FC binding pockets in CauQdr2 derived from the Boltz-2 **(C)** and Proteinix **(D)** models, highlighting residues contributing to ligand interactions. **(G,H)** Predicted 5-FC binding pockets in CauMdr1.2 derived from the Boltz-2 **(G)** and Proteinix **(H)** models, highlighting residues contributing to ligand interactions. In all pocket panels, 5-FC is shown as orange sticks and interacting residues are depicted as sticks with residue labels.

Given the elevated 5-FC resistance profiles of CauQdr2 and CauMdr1.2 overexpression strains, we were prompted to explore if 5-FC could be accommodated within the central substrate binding cavities of the two transporters. We generated 5-FC bound structural models using three state-of-the-art open-source reproduction frameworks of AlphaFold3, Boltz-2, Proteinix and Chai-1 (Abramson et al., 2024; Discovery et al., 2024; Passaro et al., 2025; ByteDance AML AI4Science Team et al., 2025). We analyzed the 5 models produced from each method and observed that across five models from each method, Boltz-2 consistently yielded the highest pTM, ipTM, and pLDDT scores for both CauQdr2 and CauMdr1.2 (Figures 6A,E, respectively). A recent benchmarking study of the open-source models also suggested much Boltz to be an overall superior alternative (Xu et al., 2025). For both CauQdr2 and CauMdr1.2, proteinix also generated good pTM, ipTM, and pLDDT scores, closely matching those obtained using Boltz-2. However, Chai-1 models showed markedly lower ipTM values, indicating lower confidence in ligand placement. Based on these metrics, subsequent structural interpretation was restricted to Boltz-2 and Proteinix only, as both produced stable folds with a 12 transmembrane helix (TMH) architecture, characteristic of MFS pumps, and a cavity capable of 5-FC accommodation (Figures 6B,F). Notably, the selected models from both methods placed the ligand within the central substrate-binding pocket formed between the N- and C-terminal six-helix bundles of CauQdr2 and CauMdr1.2 (Figures 6B,F).

To gain insights into the nature of the substrate-binding pocket, all residues within 5 Å of 5-FC were mapped (Figures 6C,D for CauQdr2; Figures 6G,H for CauMdr1.2). Interestingly, despite slight differences in the predicted ligand poses, Boltz-2 and Proteinix identified overlapping sets of pocket-lining residues for both transporters.

In CauQdr2, both models revealed a polar-aromatic cage, capable of stabilizing the heterocyclic ring of 5-FC (Figures 6C,D). Several polar residues, including S120, S121, Y152, Q156 could be seen consistently positioned near 5-FC. Furthermore, aromatic residues W371 and Y368 could be seen as aromatic stabilizing elements in the Boltz-2 model (Figure 6C), with Y368 also present in the Proteinix pocket (Figure 6D). A similar pocket architecture was observed for CauMdr1.2, with strong agreement between the models, wherein a network of polar-aromatic residues, including Y158, N123, S242 could be seen (Figures 6G,H). However, in contrast to CauQdr2, both models positioned several charged residues, viz., R493, D494, and D353 near the ligand in case of CauMdr1.2, highlighting a more electrostatically enriched interaction zone (Figures 6G,H).

## 4 Discussion

The rapid global spread of multidrug-resistant *C. auris*, together with the recent emergence of pan-drug resistant isolates, is deeply concerning (Jacobs et al., 2022; Wake et al., 2024). Besides selecting for intrinsic or acquired resistance mechanisms, antifungal monotherapy is intrinsically prone to inducing adaptive mechanisms like heteroresistance and antifungal tolerance, which can promote long-term persistence and lead to treatment failure (Wake et al., 2024). Antifungal combination therapy therefore represents a promising alternative strategy with the potential to prevent resistance development. Beyond its established role as the key therapeutic option for cryptococcal meningitis, 5-FC also serves as a combination partner for the treatment of invasive candidiasis (Sprute et al., 2023; Wake et al., 2024). Interestingly, *in vitro* studies have demonstrated strong efficacy of 5-FC against *C. auris* when used in combination with agents from all other major antifungal classes (Bidaud et al., 2019; O’Brien et al., 2020; John et al., 2023).

Since resistance threats invariably accompany the use of any antifungal agent, it is essential to develop a detailed understanding of the factors contributing to resistance. Efflux-mediated 5-FC resistance in *C. auris* has remained largely unexplored, with the exception of a recent study reporting differential regulation of two DHA1 family genes, *QDR2* and *MDR1.2*, in a strain adapted to 5-FC tolerance (Phan-Canh et al., 2025). Although the study also highlighted altered expression of two additional transporter genes *OPT2.3* and *SIT1.18*, these transporters primarily mediate the import of specialized substrates, such as oligopeptides and siderophores, respectively (Heymann et al., 2002; Dunkel et al., 2013). We therefore focused our investigation on DHA1 transporters, given their established involvement in 5-FC export and resistance, as documented in other fungi (Costa et al., 2013a; Pais et al., 2016).

### 4.1 Functional role of CauQdr2 and CauMdr1.2 in antifungal resistance

To functionally investigate the roles of these two DHA1 transporters genes, we overexpressed them as GFP-fusion proteins in a well-established heterologous system that allows high-level plasma membrane efflux pump expression. Both CauQdr2 and CauMdr1.2 were expressed, targeted to the cell membrane and conferred increased resistance to 5-FC when assessed on both liquid and solid media. Notably, the >2-fold increase in IC_50_ values observed for both CauQdr2 and CauMdr1.2 is particularly significant given that the growth medium contained a low concentration of uracil (0.002%) to support the auxotrophic host strain. Since 5-FC enters via the pyrimidine salvage pathway and is converted to 5-FU, exogenous uracil could potentially modulate pathway activity through feedback regulation or metabolic competition, thereby influencing 5-FC uptake or conversion. However, given that uracil was uniformly present in all strains, the IC_50_ shift observed specifically in the transporter-overexpressing strains is unlikely to be solely due to metabolic effects and instead supports a transporter-dependent contribution to altered 5-FC susceptibility. Notably, while the magnitude of resistance observed is modest, it is consistent with the contribution of individual transporters and likely reflects only one component of a multifactorial resistance landscape as demonstrated in *C. glabrata* (Pais et al., 2016). Interestingly, Aqr1, a close relative of Qdr group of transporters, has previously been implicated in 5-FC transport in *C. glabrata* (Costa et al., 2013a), lending further support to the involvement of CauQdr2 in 5-FC transport. In contrast, very little is known about CauMdr1.2 and its role. Although annotated as a homolog of Mdr1 from *C. albicans*, its close homolog in *S. cerevisiae* is Yhk8 (Khatoon et al., 2022b), a transporter not previously demonstrated to function as a multidrug efflux pump, though it is transcriptionally upregulated in azole-resistant laboratory-adapted strains (Barker et al., 2003). Phylogenetic analysis of yeast DHA1 transporters places CauMdr1.2 outside the Flr1/Mdr1 clade (Khatoon et al., 2022b). Consistently, our comparative phylogenetic analysis of known 5-FC transporters also placed CauMdr1.2 as an outgroup arguing against its classification as a second homolog of Mdr1 in *C. auris*. With respect to functional classification, our data suggests that CauMdr1.2 differs functionally from classical Mdr1-type transporters. CauMdr1.2 overexpression did not confer resistance to known Mdr1 substrates, including azole drugs such as fluconazole, voriconazole, and clotrimazole and other xenobiotics like benomyl, 4-NQO, etc.

A striking observation was also noted for CauQdr2, where we observed that the overexpression strain exhibited increased sensitivity to quinidine compared to the host strain, thereby excluding the possibility of quinidine acting as an efflux substrate. In *C. albicans*, none of the Qdr homologs have been implicated in quinidine resistance, however, the corresponding overexpression strains also did not display any enhanced sensitivity (Shah et al., 2014). The observed quinidine sensitivity in AD-CauQdr2 could be either due to alterations at the level of plasma membrane or from intracellular stress associated with transporter overexpression. *QDR* genes are known to participate in lipid and cation homeostasis as well as oxidative stress response in yeasts, and similar roles may be conserved in *C. auris QDR2* as well (Vargas et al., 2007; Ríos et al., 2013; Shah et al., 2014). On the contrary, although direct transport of quinidine was not assessed in this study, a potential involvement of CauQdr2 in quinidine import appears more plausible, particularly given the selective sensitivity observed toward quinidine and not other antifungals tested. Notably, increasing evidence supports the role of MFS pumps in drug import (Galocha et al., 2020). For instance, the siderophore transporter Sit1 in *Aspergillus fumigatus* and *C. glabrata* mediates uptake of the antifungal VL-2397 (Dietl et al., 2019), while Sit1 from *C. albicans* has recently been shown to facilitate caspofungin uptake (Pedras et al., 2026). Our group has also demonstrated the involvement of a *C. auris* peptide permease, Ptr_C, in the uptake of antifungal peptides such as Nva-FMDP and Nikkomycin Z (Khatoon et al., 2022a, 2025). Notably, several DHA1 transporters have been reported to function as importers of antifungal compounds. *C. albicans* Mdr1 has been shown to import the antifungal small molecule, bis[1,6-a:5′,6′-g]quinolizinium 8-methyl-salt (BQM) and the cyanine derivative MKT-077 (Sun et al., 2013; Chen et al., 2026), and Hol1 has recently been implicated in ATI-2307 uptake (Karadzas et al., 2025). A detailed characterization of the plausible importer specific attributes of CauQdr2 could open new avenues for antifungal drug design.

Notably, both *MDR1.2* and *QDR2* were demonstrated to be differentially regulated in amphotericin B-resistant clinical isolates, with the former transcriptionally activated and the latter repressed (Shivarathri et al., 2022). Given these observations, we also evaluated the susceptibility of the overexpression strains toward amphotericin B but observed no increase in resistance relative to the host strain. Collectively, our antifungal screening data indicates that both CauQdr2 and CauMdr1.2 most likely recognize 5-FC as their substrate, supporting a specific role for DHA1 transporters in 5-FC resistance in *C. auris*.

### 4.2 Clorgyline as a selective efflux inhibitor

Combination therapy incorporating efflux inhibitors represents an effective strategy to sensitize resistant strains to antifungals, thereby increasing the lifespan and efficacy of antifungals. With this rationale, we evaluated the ability of the fungal ABC and MFS efflux pump inhibitor clorgyline (CLO) to sensitize the overexpression strains to sub-inhibitory doses of 5-FC. Notably, synergistic activity was observed exclusively in the AD-CauMdr1.2 strain, where a discrete 3×3 concentration window yielded Bliss synergy scores >10, indicative of a synergistic interaction between 5-FC and CLO. While we observed that CauMdr1.2 does not share the characteristic substrate profile of Mdr1 homologs, the capacity of CLO to reduce the magnitude of 5-FC resistance indicates some functional overlap. While CLO’s synergistic ability is well documented with azole drugs in *Candida* species and recombinant strains overexpressing Cdr1 and Mdr1 transporters (Holmes et al., 2012; Toepfer et al., 2023a), it has been reported to decrease, rather than enhance, susceptibility to other antifungal classes such as echinocandins and polyenes, including micafungin and amphotericin B (Nagayoshi et al., 2017). Conversely, enhanced 5-FC activity in the presence of CLO has been described in *A. fumigatus* (Sastré-Velásquez et al., 2022). These contrasting observations further strengthen the argument that CLO’s synergistic effects are primarily attributable to efflux inhibition, as echinocandins and polyenes are not part of the efflux arsenal of fungal drug transporters (Cannon et al., 2009; Holmes et al., 2016). Despite the fact that CLO can modulate efflux abilities of distinct classes of drug transporters (Toepfer et al., 2023a), the selective activity of CLO with CauMdr1.2, even though CauQdr2 is also a DHA1 pump, highlights a notable degree of specificity in its inhibitory action. However, as these transporters were analyzed in an overexpression-based heterologous system, the possibility of off-target or indirect effects cannot be fully excluded, including potential transcriptional modulation of resistance determinants. Although the mechanism of CLO’s inhibitory activity remains poorly understood, it most likely involves interaction with drug-binding site(s), a hypothesis that will require detailed structural and biochemical analyses to resolve.

### 4.3 Structural insights into 5-FC recognition

While biochemical and structural studies have provided important insights into substrate binding and transport by fungal DHA1 pumps (Redhu et al., 2016b, 2018; Banerjee et al., 2021; Alves et al., 2023), the mode of interaction of 5-FC with these efflux pumps has remained unexplored. In this study, we therefore mapped the putative 5-FC-binding sites of CauQdr2 and CauMdr1.2, using structural models, with the aim of identifying residues that may potentially contribute to 5-FC recognition. Notably, the pocket architecture mirrors the substrate-binding pockets of other prototypical MFS multidrug transporters like MdfA, which exploit mixed polar–hydrophobic/aromatic microenvironments to accommodate chemically and structurally diverse xenobiotics (Heng et al., 2015). Interestingly, some residues forming the predicted pockets localize to conserved MFS motifs, including motifs D2, A, B, C, and G (Table 2). Although the residues listed in the table fall strictly within these motifs, additional interactive residues were identified in adjoining regions. For instance, S242 and V243 in CauMdr1.2 immediately precede the antiporter motif (motif C). Previous mutational analyses of CaMdr1 and the *Staphylococcus aureus* tetracycline/H^+^ antiporter TetA(K) have highlighted several residues both within and flanking this motif to be important for substrate transport (Ginn et al., 2000; Pasrija et al., 2007). As expected, Motif A located in a cytoplasmic loop and involved in conformational changes during transport (Sharma et al., 2022), did not contribute directly to the predicted substrate interaction region. Interestingly, S211 in CauQdr2 corresponds to a highly conserved residue within motif B of fungal DHA1 pumps and alanine substitution of the equivalent residue in CaMdr1 renders *S. cerevisiae* cells drug-susceptible. Motif B also harbors the critical arginine required for proton translocation in CaMdr1 (Redhu et al., 2016a). Furthermore, alanine substitution of a few residues with motifs D2 and G have previously been shown to be debilitating for CaMdr1 function (Redhu et al., 2018; Sharma et al., 2022).

**Table 2:** Motif-derived residues contributing to the predicted flucytosine (5-FC) interaction zones of CauQdr2 and CauMdr1.2.

Motif name	Consensus sequence	Location	Motif residues in the 5-FC interaction zone (CauQdr2)	Motif residues in the 5-FC interaction zone (CauMdr1.2)
Motif D2	lgxxxxxPvxP	TMH1	S124, P125	T126
Motif A	GxLaDrxGrkxxxI	Cytoplasmic loop between TMH2 and TMH3	–	–
Motif B	IxxxRxxqGxgaa	TMH4	S211	–
Motif C	gxxxGPxxGGxI	TMH5	Q245	S246
Motif G	GxxxGPL	TMH11		A497

Included residues are part of the Boltz-2 and/or Proteinix structural models and reside within the defined motif. Motif annotations follow the conserved Major Facilitator Superfamily (MFS)/DHA1 signature regions described by Putman et al. (2000). A residue is considered part of the 5-FC interaction zone if any side-chain atom is within 5 Å of the 5-FC molecule in the selected models.

The available comprehensive alanine-scanning mutagenesis data for the transmembrane helices of CaMdr1 (Redhu et al., 2018) enabled comparison of residues highlighted in the 5-FC–bound structural models of CauQdr2 and CauMdr1.2 with functionally characterized positions in CaMdr1. Strikingly, for both transporters, most residues predicted to constitute the binding pocket, when mapped to their equivalent positions in CaMdr1, conferred complete susceptibility to all tested antifungal agents upon substitution to alanine (or to glycine when alanine is the native residue), underscoring their critical role in drug recognition and transport (Supplementary Table S3). For CauQdr2, these positions correspond to S120, S121, Y152, S153, Q156, P160, Q245, Y368, W371, L372, L375, and R510, whereas for CauMdr1.2, such positions were mapped to N123, M155, Y158, S246, D353, F357, Y467, R493, and A497. Collectively, these findings are consistent with the possibility that the identified residues may contribute to a putative 5-FC interaction region in both transporters and potentially to the recognition of other substrates. Although experimental validation through targeted mutagenesis is still required, our analysis provides a structural template to guide future functional studies of 5-FC transport by CauQdr2 and CauMdr1.2.

### 4.4 Limitations

In this study, we employed a gain-of-function expression system which may potentially amplify physiological effects. We opted for this approach instead of constructing gene deletion mutants because the host strain lacks major endogenous efflux pumps, thereby minimizing background interference. Indeed, our previous work demonstrated that *C. auris MDR1* deletion phenotypes were masked due to the presence of dominant compensatory determinants, whereas overexpression in this heterologous system made phenotypes such as resistance to antifungal azoles evident (Khatoon et al., 2022b). While endogenous overexpression or targeted gene deletion in *C. auris* would provide valuable in-species genetic validation, the known functional redundancy of efflux systems makes phenotypic interpretation challenging; therefore, the heterologous system serves as a robust surrogate for initial functional assessment.

Furthermore, although growth-based susceptibility assays with antifungal drugs suggest a transport route for 5-FC, our study does not directly measure 5-FC transport, and thus definitive evidence of 5-FC efflux remains to be established. Future studies employing direct transport assays using radiolabeled substrate or intracellular drug quantification will be required to validate the proposed mechanism. While the structural models generated provide useful predictive insights into potential mode of 5-FC recognition, their computational nature necessitates experimental validation. The same limitation applies to the proposed mechanism of CLO-mediated sensitization of CauMdr1-overexpressing *S. cerevisiae* toward 5-FC. Direct substrate binding assays and transport measurements will be required to confirm CLO interaction with CauMdr1 and to delineate its modulatory effects on 5-FC transport.

## Data Availability

The original contributions presented in the study are included in the article/Supplementary material, further inquiries can be directed to the corresponding author.
