# Supplementary material for: Functional characterization of Candida auris DHA1 transporters conferring flucytosine resistance
Source: Front Microbiol. 2026 May 8;17:1806653. doi: 10.3389/fmicb.2026.1806653 (PMC13194421; doi:10.3389/fmicb.2026.1806653)
Supplement: Supplementary file 1 [file Supplementary_Tables.DOCX]

**Supplementary tables**

**Supplementary table S1: List of oligonucleotides used in the study**

| **S. No.** | **Primer ID** | **Sequence (5'-3')** |
| --- | --- | --- |
| **Cloning primers** | | |
| 1. | 2663_PacI | CGCGATTAATTAAATGGAGACTCCAGACGAGGAATTAG |
| 2. | 2663_NotI | CGCGAGCGGCCGCAATTGTGTGTGTACAAGTACACG |
| 3. | 4113_PacI | CGCGATTAATTAAATGGACAGAGAAATATCTCGGG |
| 4. | 4113_NotI | CGCGAGCGGCCGCATACATCCTCCTTCTCAGTCTC |

**Supplementary table S2: List of strains used in the study**

| **S. No.** | **Strain ID** | **Description** | **Reference** |
| --- | --- | --- | --- |
| 1. | AD1-8u^‒^ | *MATα PDR1–3 ura3 his1* Δ*yor1*::*hisG* Δ*snq2*::*hisG* Δ*pdr10*::*hisG* Δ*pdr11*::*hisG* Δ*ycf1*::*hisG* Δ*pdr3*::*hisG* Δ*pdr5*::*hisG* Δ*pdr15::hisG* | (Lamping et al., 2007) |
| 2. | AD-CauQdr2 | AD1-8u^‒^, Δ*pdr5*::pABC3- CauQdr2-GFP | This study |
| 3. | AD-CauMdr1.2 | AD1-8u^‒^, Δ*pdr5:*:pABC3- CauMdr1.2-GFP | This study |

**Supplementary table S3: 5-FC–interacting residues in CauQdr2 and CauMdr1.2 structural models and reported mutational phenotypes in CaMdr1.**

Residues predicted to contribute to the 5-FC interaction zone in CauQdr2 and CauMdr1.2 were mapped to equivalent positions in CaMdr1 using multiple sequence alignment. The table summarizes antifungal susceptibility data of alanine substitution mutations (or glycine substitutions when alanine is the native residue) at these positions in CaMdr1 as reported by (Redhu et al., 2018).*TS: susceptibility to all the tested drugs;* *SS: selective susceptibility to the tested drugs; NS: no susceptibility*

| **Residue Position in 5-FC-bound structural models** | | **Equivalent residue in CaMdr1** | **Antifungal susceptibility phenotype of CaMdr1 overexpressing AD1-8u^‒^ strain** |
| --- | --- | --- | --- |
| **CauQdr2** | S120 | V130 | TS |
|  | S121 | Y131 | TS |
|  | S124 | S134 | NS |
|  | P125 | A135 | NS |
|  | Y152 | F162 | TS |
|  | S153 | V163 | TS |
|  | Q156 | Y166 | TS |
|  | P160 | P170 | TS |
|  | S211 | S223 | SS |
|  | I214 | L226 | NS |
|  | L242 | V254 | NS |
|  | Q245 | P257 | TS |
|  | Y368 | Y365 | TS |
|  | W371 | L368 | TS |
|  | L372 | Y369 | TS |
|  | L375 | F372 | TS |
|  | N507 | D501 | NS |
|  | R510 | R504 | TS |
|  | C511 | S505 | SS |
| **CauMdr1.2** | N123 | Y131 | TS |
|  | T126 | S134 | NS |
|  | M155 | V163 | TS |
|  | Y158 | Y166 | TS |
|  | S242 | A253 | NS |
|  | V243 | V254 | NS |
|  | S246 | P257 | TS |
|  | D353 | Y365 | TS |
|  | F357 | Y369 | TS |
|  | Y467 | F477 | TS |
|  | G490 | D501 | NS |
|  | R493 | R504 | TS |
|  | D494 | S505 | SS |
|  | A497 | A508 | TS |
